# A National Survey of Prehospital Care Services of United Kingdom for Use, Governance and Perception of Prehospital Point of Care Ultrasound

**DOI:** 10.24908/pocus.v7i2.15739

**Published:** 2022-11-21

**Authors:** Salman Naeem, Christopher Edmunds, Thomas Hirst, Julia Williams, Amir Alzarrad, James Ronaldson, Jon Barrat, East Anglian Air Ambulance Research, Audit, Innovation and Development (RAID) Group, PreHospital Trainee Operated Research Network (PHOTON)

**Affiliations:** 1 Barts Health NHS trust London United Kingdom; 2 East Anglian Air Ambulance Norwich United Kingdom; 3 Adult Critical Care Transfer Service Cymru; 4 South East Coast Ambulance NHS Foundation Trust United Kingdom; 5 Scottish Ambulance service, ScotSTAR United Kingdom; 6 Royal College of Surgeons Edinburgh United Kingdom

**Keywords:** PoCUS, HEMS, Trauma, Pre-hospital, ultrasound, governance

## Abstract

**Introduction: **Point of care ultrasound (POCUS) has become a common practice in prehospital care over the last 10 years. There is lack of literature on its use and governance structure in United Kingdom (UK) prehospital care services. We aimed to survey the use, governance of prehospital POCUS among UK prehospital services and perceptions of clinicians and services regarding its utility and barriers to its implementation. **Methods: **Four electronic questionnaire surveys were delivered to UK helicopter emergency medical service (HEMS) & clinicians, ambulance and community emergency medicine (CEM) services between 1^st^ of April and 31^st^ of July 2021 investigating current use, governance structure for POCUS and perception about its benefits and barriers. Invitations were sent via email to medical directors or research leads of services and using social media. Survey links remained live for two months each. **Results: **Overall, 90%, 62% and 60% of UK HEMS, ambulance and CEM services respectively, responded to surveys. Most of the services used prehospital POCUS, however only two HEMS organisations fulfilled the Royal College of Radiology governance criteria for POCUS. The most commonly performed POCUS modality was echo in cardiac arrest. Majority of clinicians judged POCUS to be beneficial and the common perceived benefit was promotion of enhanced and effective clinical care. Major barriers to its implementation included a lack of formal governance, limited literature supporting its use and difficulties in performing POCUS in prehospital environment. **Conclusion: **This survey demonstrates that prehospital POCUS is being provided by a majority of the prehospital care services and clinicians have found it beneficial in providing enhanced clinical care to their patients. However, the barriers to its implementation are relative lack of governance structure and supportive literature.

## Introduction

Point of care ultrasound (POCUS) was identified in 2011 as one of the top five research topics relevant to prehospital clinical practice 1. This statement defined three key questions for research to focus on. 1. Which ultrasound examinations can be reliably transferred to the pre-hospital setting? 2. How does prehospital ultrasound affect patient management and the patient pathway? 3. How should providers achieve and maintain specific ultrasound skills?

This statement was in part in response to the sharp rise of POCUS use within in-hospital care settings, where its use has been shown to improve diagnostic accuracy having a valuable impact on patient care 2, 3, 4, 5, 6. Naturally, as equipment and technology has improved specifically with consideration to cost and size of equipment, it has become possible for prehospital clinicians to take ultrasound capability with their teams to patients at the scene of incident. Importantly, the image quality has improved despite ultrasound devices becoming smaller and more portable 7.

Despite these advancements in accessibility, the literature showing mortality benefit is limited 8, 9. Prehospital services in North America, Europe, and Australia have been able to demonstrate some improvements in on-scene diagnostic accuracy 10, 11. There have been several systematic reviews examining POCUS training in the prehospital setting, which demonstrated a large variety of training approaches ranging from very short to much longer processes. What is clear is that there is no overarching standard set out for prehospital clinicians and limited routes to formal accreditation for POCUS use in the pre-hospital setting. The existing data suggest that a mixed practical and online learning training package maybe the best approach. It is also important to consider that the level of clinician being trained in ultrasound use ranges from junior to senior with varying degrees of success 12, 13, 14.

To truly understand and answer the research questions defined in 2011, we felt it was important to understand how POCUS is used in current UK pre-hospital practice. This survey aimed to assess prehospital POCUS use, governance and perception about its benefits and barriers to implementation by the UK Helicopter Emergency Medicine Services (HEMS), HEMS clinicians, national ambulance services and Community Emergency Medicine (CEM) services. 

## Methods

A research group consisting of prehospital clinicians with a specific interest in POCUS from across the UK was formed. The aim of the group was to determine a method to better understand the use of POCUS in UK prehospital setting. The group determined that it was important to both understand and assess the individual clinician and service level perspectives of prehospital POCUS use. Key areas of question were identified, specifically barriers and benefits to use, training requirement, and implementation of prehospital POCUS governance. A series of questionnaires were developed with questions structured using Likert scales, yes/no answers, and ranking questions. The target groups were defined by their scope of work in prehospital environment – UK HEMS, HEMS clinicians, ambulance service and CEM service. 

Four final surveys were developed, three tailored to each type of target organization and one for the HEMS clinicians. The questionnaires were designed with mandatory questions to ensure data entry prior to submission. Eight core questions were defined across the services surveys so a comparison could be made between the different settings. The surveys were hosted on Google Forms which remained live for two months each. The total data collection period was between 1^st^ of April to 31^st^ of July, 2021. They were distributed electronically, and responses were further directly sought via email to clinical or research leads of specific organizations for services survey. Clinical leads were asked to get response from their clinicians via email and social media posts were shared online to try and capture as many active HEMS and individual clinicians as possible. Duplicate entries were eliminated using Google Forms, and first response was considered for final analysis. Descriptive analysis of the results was undertaken using Microsoft Excel and all data were kept anonymous.

The surveys met the UK National Institute for Health and Care Research (NIHR) criteria as service evaluation; hence a formal Health Research Authority approval was not required 15. The Checklist for Reporting Results of Internet E-Surveys (CHERRIES) had been followed in reporting this survey 16.

## Results

Responses were obtained for all 4 surveys from 19 of 21(90%) UK HEMS organisations, 8 of 13 (62%) NHS Ambulance trusts and 3 of 5 (60%) CEMS organisations. A majority of the HEM services (17) catered to both urban and rural population while most of the ambulance and CEM services (7 and 2 respectively) provided services to rural population. The median transfer interval for HEMS, ambulance and CEMS were 26.2, 24.5 and 15 minutes respectively. POCUS was being provided by doctors, nurses and allied health professional in the HEMS and CEM organisations and by critical care/advanced paramedics in the ambulance services. 

**Figure 1:**
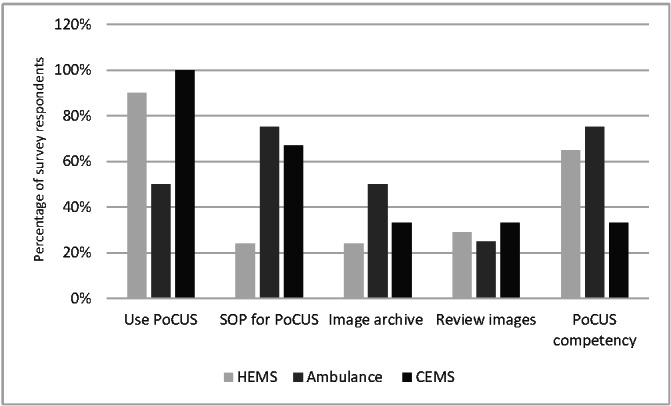
Clustered column chart showing the use and aspects of governance surveyed for prehospital POCUS. POCUS, point-of-care ultrasound; SOP, standard operating procedure; HEMS, helicopter emergency medical services; CEMS, community emergency medical services.

**Figure 2:**
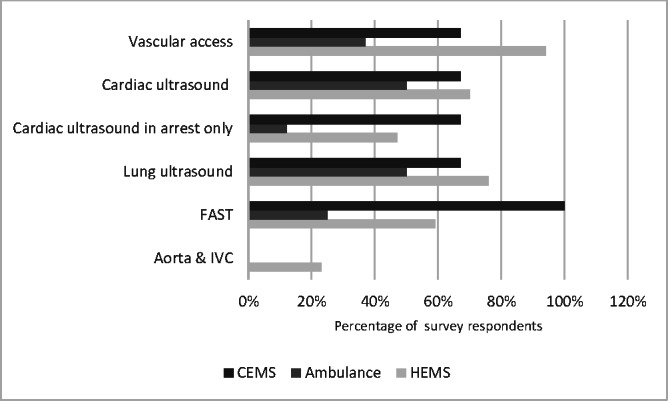
Clustered bar chart depicting modalities of POCUS-provided pre-hospital care services. POCUS, point-of-care ultrasound; FAST, focused assessment with sonography in trauma; IVC, inferior vena cava; HEMS, helicopter emergency medical services; CEMS, community emergency medical services.

POCUS was being provided by 17 of the 19 HEMS organisations, 4 of the 8 ambulance services and 3 of the 3 CEMS organisations in UK. The results about the use and governance standards are given in Figure 1. The modalities of POCUS provided by HEMS, ambulance and CEMS are shown in Figure 2. Lack of evidence for benefit, accepted standards and equipment not suited for prehospital environment were highlighted as significant barriers to the use of POCUS by ambulance and CEM services. Further details are available in supplementary Table S1.

There were 265 respondents from 20 air ambulance organisations nationally, out of which 176 (66%) were doctors and 89 were (34%) AHP & nurses. Basic demographics are depicted in Table 1. The replying doctors included 124 consultants and 51 registrars, 93 working in emergency medicine (53%), 43 in anaesthetics (26%) and 24 in intensive care medicine (14%). Responses from AHP & nurses included 53 critical care paramedics (57%) and 34 HEMS paramedics (35%). Considering training, 57 (32%) of doctors and 76 (85%) of AHPs & nurses held no formal ultrasound accreditation. 

**Table 1:** Demographics of HEMS clinicians.

	**Total**	**Doctors**	**AHPs**
**HEMS Clinicians**	265	176	89
**Age**			
<20 years	1	1	0
20-29 years	9	0	9
30-39 years	118	93	25
40-49 years	114	70	44
50-59 years	22	11	11
60-69 years	1	1	0
70-79 years	0	0	0
**Sex**			
Male	221	144	77
Female	43	31	12
Prefer not to say	1	1	0
**Pre-hospital Role**			
Doctor Consultant		124	
Doctor Registrar		51	
Critical Care Paramedic		53	
HEMS Paramedic		34	
Specialist Nurse		1	
Others		2	

**Figure 3:**
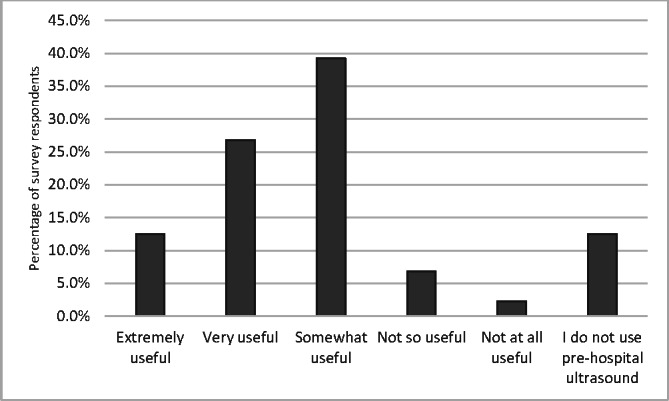
Clustered column chart showing perceived utility of prehospital POCUS by HEMS clinicians. POCUS, point-of-care ultrasound; HEMS, helicopter emergency medical services.

**Figure 4:**
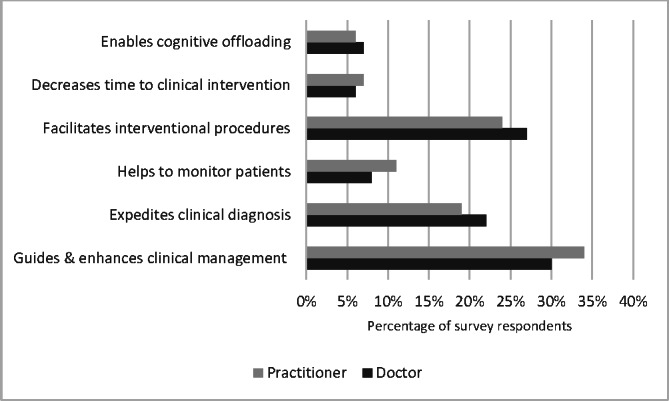
Clustered bar chart showing perceived benefits of pre-hospital POCUS by HEMS clinicians. POCUS, point-of-care ultrasound; HEMS, helicopter emergency medical services

**Figure 5:**
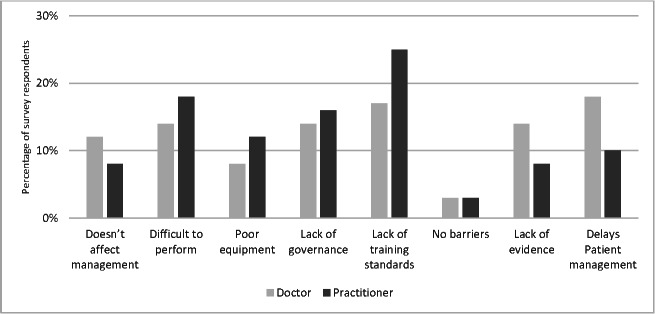
Clustered column chart showing perceived barriers to prehospital POCUS by HEMS clinicians. POCUS, point-of-care ultrasound; HEMS, helicopter emergency medical services.

The most commonly used ultrasound modalities by the clinicians in the prehospital environment were echocardiography in life support (ELS), lung ultrasound and vascular access. Ultrasound was considered somewhat useful by 104 respondents (39%) and very useful by 71 respondents (27%) as showin in Figure 3 The major benefits of POCUS were informing clinical management, namely expediting diagnosis and facilitating clinical interventions. The barriers to POCUS were lack of evidenced benefit, lack of governance and challenges in performing studies in the pre-hospital environment. The summary of perceived benefits of and barriers to POCUS in the pre-hospital environment are detailed in Figures 4 and 5 with further details in supplementary Table S2. 

## Discussion

This is the first UK national survey delivering a snapshot of UK prehospital POCUS practice. POCUS is provided by 100%, 90% and 33% of the CEMS, HEMS and ambulance services that responded. This is a higher national proportion compared to European and American counterparts. A 2014 North American cross-sectional convenience survey shows that 4.1% of Emergency Medical Services (EMS) incorporate ultrasonography within their prehospital care 10. A one-month multi-national survey in 2020 reported 75% of European HEMS services were using a POCUS assessment 17. This comparison may not accurately reflect the current state of POCUS use due to lack of robust studies and data. This data highlights that standardisation of POCUS use, formal clinician accreditation processes and governance systems are not universally employed, and different protocols are used for the assessment of patients 18.

The data suggests that the most common prehospital POCUS scans performed are cardiac ultrasound, lung ultrasound, vascular access and FAST. This correlates with previous studies conducted internationally which have shown FAST to be the most common ultrasound study undertaken by HEMS in Europe and elsewhere  17, 19, 20, 21.

Formal image review and storage of scans in clinical records is uncommon and is not widely adopted. The Royal College of Radiologists (RCR) has specific guidance for the governance of POCUS^22^ which emphasise on following key principle i.e written guidance (SOP) for ultrasound examinations taken, appropriate training and accreditation of the clinicians, quality assurance of practice, archiving of images and inclusion of images in patient’s records. Our data shows that the majority of organisations do not yet fulfil the RCR requirements for the governance of POCUS. These results demonstrate the lack of local POCUS governance systems. This is at odds to the typical delivery of prehospital care where governance plays a central role  23.

Credentialing aims allow achievement of a minimum standard in POCUS ability ensuring that clinicians all have a baseline standard of practice. The data highlights that 32% (57) of doctors and 85% (76) of AHP’s respondents were not formally accredited. These figures may be influenced by the availability of accreditation within a specialty or professional role. It is important to recognise that clinicians may have had sufficient training in POCUS to be safe and competent without being formally accredited. The majority of services using POCUS have a competency pathway and most of them rely on “sign off” process from outside bodies i.e, Royal College of Emergency Medicine (RCEM), Focused Ultrasound for Intensive Care (FUSIC). Importantly, the survey highlights the lack of training and quality assurance as a barrier for prehospital POCUS and the need for an improved national governance framework including image storage and review.

Prehospital POCUS is yet to evidence mortality benefit for patients. In many studies there are perceived benefits, allowing early diagnosis and decreasing time to clinical interventions 17. There has been evidence found of a beneficial association between patient care through early intervention and expedited diagnosis in-hospital POCUS  6, 8, however, there is paucity of evidence in the prehospital field. A number of studies have highlighted possible patient harm by delaying CPR in cardiac arrest or increasing pre-hospital scene times, adding to delay in patient transfer 17, 20. This survey scrutinises perceptions of prehospital clinicians and services about the benefits and barriers to prehospital POCUS. Most clinicians (78%) judge it useful for patient management, helping to enhance clinical management, expediting diagnosis and facilitating interventions. These results are similar to other studies about utility of POCUS in hospital settings while the major barrier to POCUS use is lack of training and equipment 24, 25, 26, 27. This has been recognised by national speciality colleges by incorporating POCUS into training curricula as a required skill. 

## Limitations

This is an online survey-based study and our response rate from UK ambulance and CEM services of only 62% and 60%, hence it may not fully represent the practice of all services. Similarly, the increased likelihood of response from HEMS clinicians with a developed ultrasound practice risks selection and response bias. Only 265 of HEMS clinicians responded so this may not accurately represent the opinions and practice of all HEMS clinicians nationally. National BASICS and other prehospital clinical providers, such as search and rescue, motorsport and mountain rescue organisations were not included which meant that data may not be fully representative of all UK pre-hospital medical practice. 

## Conclusion

This study is the most comprehensive current evaluation of UK pre-hospital POCUS use and gives useful insight into perceptions of its utility and barriers to implementation of pre-hospital POCUS. It demonstrates that pre-hospital POCUS is feasible and is being currently utilised by most UK prehospital services. The major barrier to its use is lack of governance and literature supporting its use in prehospital setting. It also highlights the need for clear prehospital POCUS guidance from national governing bodies to ensure this important clinical tool is utilised safely and effectively with strong governance processes in place. More research is required to assess the utility and benefits of POCUS in the UK pre-hospital settings. 

## Funding

There is no funding provided in conducting or publication of this survey.

## Disclosure statement

The authors report there are no competing interests to declare.

## Availability of data and materials

The anonymised datasets generated and/or analysed during the current study are not publicly available due confidentiality but are available from the corresponding author on reasonable request.

## Supplementary Material

Supplementary Table S1 Prehospital services survey results.

Supplementary Table S2 HEMS clinicians survey results.
