# Supplementary material for: A National Survey of Prehospital Care Services of United Kingdom for Use, Governance and Perception of Prehospital Point of Care Ultrasound
Source: POCUS J. 2022 Nov 21;7(2):232–8. doi: 10.24908/pocus.v7i2.15739 (PMC9983728; doi:10.24908/pocus.v7i2.15739)
Supplement: Supplementary Table S1 [file pocusj-07-15739-s001.pdf]

POCUS Journal 2022; https://doi.org/10.24908/pocus.v7i2.15739

Supplementary Table S1: Prehospital services survey results.

Abbreviations: PoCUS, point-of-care ultrasound. SOP, standard operating procedure. HEMS, helicopter emergency medical services. CEMS, community emergency medical services, IVC, inferior vena cava

Prehospital services survey results (Table 1)

Question/Answers	Total Responses		
	**HEMS Services**	**Ambulance Service**	**CEM Services**
**Service respondents?**	19	8	3
**How many pre-hospital clinicians are employed by your service including doctors, paramedics, and nurses? or How many advanced paramedics does your service employ? (Ambulance service survey)**	42 (12-67)	116 (33-394)	16 (10-20)
**Which paramedics use pre-hospital ultrasound? (Ambulance service survey only) or Which Clinicians use ultrasound in your service? (CEM)**			
None	N/A	3	N/A
Critical Care Paramedic	N/A	4	N/A
Advanced/Specialist Paramedic	N/A	1	N/A
All	N/A	N/A	2
Doctors Only	N/A	N/A	1
**Which population does your service cater to?**			
Both	17	1	1
Urban	1	0	0
Rural	1	7	2

**What is your median transfer time from scene to hospital and how many jobs a year?**			
Mean number of jobs per year (Ambulance survey specialist paramedics taskings only)	1773.8 (400-3680)	5000-343168	3000 (2000-4000)
Mean median transfer time	26.2 (12-49)	24.5 (16-52)	15 (10-20)
**Does your HEMS service use pre-hospital point of care ultrasound?**			
Yes	17	4	3
No, don't use ultrasound	2	4	0
**Does your service have a standard operating procedure describing the accepted use of pre-hospital ultrasound?**			
Yes	4	3	2
No	13	1	1
N/A, as we don't use ultrasound	2	4	N/A
**Does your service include/save ultrasound images in its clinical record?**			
Yes	4	2	1
No	13	2	2
N/A, as we don't use ultrasound	2	4	N/A

**If yes are these images reviewed for governance of ultrasound images? (Specifically, by individuals trained at supervisor level for point of care ultrasound)**			
Yes	5	1	1
No	12	3	2
N/A, as we don’t use ultrasound	2	4	N/A
**Does your service have a described individual clinical standard for pre-hospital ultrasound, via a sign off process or equivalent?**			
Yes	11	3	1
No	6	1	2
N/A	2	4	N/A
**If yes do your clinicians have to show competency via a sign off process or have to show equivalence of an equivalent standard or training in ultrasound?**			
Sign off	6	1	1
Equivalent Training	2	1	N/A
Equivalent Certification	2	1	N/A
N/A	2	4	N/A
No	6	1	2

Yes	1	N/A	N/A
**How many pre-hospital ultrasound studies does your service undertake each year?**	138.8 (75-300) (9 services only)	166 (100-300)	300 (100-500)
**Which modalities are used within your service?**			
Vascular access	16	3	2
Cardiac ultrasound	12	4	2
Cardiac ultrasound in arrest only	8	1	2
Lung ultrasound	13	4	2
Focused abdominal sonography in trauma	10	2	3
Aorta and IVC ultrasound	4	0	3
N/A, as we don't use ultrasound	2	4	0
**What are the perceived barriers your service is facing in introducing pre-hospital ultrasound?**			
It does not affect pre-hospital clinical management	N/A	0	
Difficult to perform in an outdoor environment	N/A	1	
The equipment is not suited for pre-hospital environment e.g heavy, poor quality, multiple probes	N/A	4	1
It delays patient care	N/A	3	

There is lack of governance including training for pre-hospital ultrasound	N/A	3	
There is lack of accepted standards and accreditation in pre-hospital ultrasound	N/A	3	1
There is lack of evidence to show benefit of pre-hospital ultrasound	N/A	4	
There are no barriers to use of pre-hospital ultrasound	N/A	0	1
**Do you see your service adopting pre-hospital ultrasound in future? (Ambulance and CEM service)**			
Yes	N/A	1	N/A
No	N/A	0	N/A
Possibly	N/A	3	N/A
N/A as we already use pre-hospital ultrasound	N/A	4	3
