# Supplementary material for: A National Survey of Prehospital Care Services of United Kingdom for Use, Governance and Perception of Prehospital Point of Care Ultrasound
Source: POCUS J. 2022 Nov 21;7(2):232–8. doi: 10.24908/pocus.v7i2.15739 (PMC9983728; doi:10.24908/pocus.v7i2.15739)
Supplement: Supplementary Table S2 [file pocusj-07-15739-s002.pdf]

POCUS Journal 2022; https://doi.org/10.24908/pocus.v7i2.15739

Supplementary Table S2: HEMS clinicians survey results.

Abbreviations: ELS, echocardiography in life support. RCEM: royal college of emergency medicine. FICE, focused intensive care echo. FUSIC, focused ultrasound in intensive care. FAST, focused assessment with sonography in trauma, FATE, focused assessed transthoracic echo. FEEL, focused echocardiography in emergency life support. FAMUS, focused acute medicine ultrasound. BSE, British society of echocardiography. RCR, royal college of radiology

HEMS Clinician Survey Results

Question/Answers	Total Responses		
	Overall	Doctor	AHP
**Which pre-hospital service do you work for?**	265	176 (66%)	89 (34%)
**Number of services represented.**			
**What is your age?**			
<20 years	1	1	0
20-29 years	9	0	9
30-39 years	118	93	25
40-49 years	114	70	44
50-59 years	22	11	11
60-69 years	1	1	0
70-79 years	0	0	0
**What is your sex?**			
Male	221	144	77
Female	43	31	12
Prefer not to say	1	1	0

**What is your pre-hospital role and grade?**			
Consultant – only if Consultant in base specialty		124	
Registrar		51	
Critical care paramedic		53	
Specialist nurse		1	
HEMS paramedic		34	
Other – free text		2	
**Do you work in another healthcare setting, if so, which?**			
Emergency medicine	95	93	2
Intensive care medicine	25	24	1
Anaesthetics	46	46	0
General practice	5	5	0
Acute Medicine	0	0	0
Surgical specialty	1	1	0
Paramedic (non-HEMS role)	51	0	51

I only work in a HEMS role	37	6	31
Cardiology	1	1	0
Other	3	0	3
Critical Care	1	0	1
**In which settings do you use point of care ultrasound (pre-hospital, hospital, both)?**			
I use ultrasound in a pre-hospital setting	51	17	34
I don't use ultrasound in a pre-hospital setting	21	2	19
I use ultrasound in a hospital or primary care setting	23	22	1
I don't use ultrasound in primary care or hospital setting	2	2	0
I only work in a pre-hospital setting and not in a hospital or primary care setting	34	4	30
Both	114	112	2
Pre-hospital not hospital	1	1	0
Hospital not pre-hospital	13	13	0
Neither	6	3	3

**Do you hold a formal accreditation in point of care ultrasound? (Attended a course, developed a logbook and been assessed as competent).**			
RCEM Level 1, 2 or Core	80	74	6
FICE or FUSIC	18	17	1
FATE	0	0	0
FEEL	11	8	3
FAMUS	0	0	0
BSE Accreditation	1	1	0
RCR Accreditation	0	0	0
I don't hold formal ultrasound accreditation	133	57	76
More than one of the above	22	19	3
**Which point of care ultrasound modalities do you use in a pre-hospital setting?**			
Echo in cardiac arrest	184	130	54
Echo in shocked or critically ill patient	87	68	19

Extended focused abdominal sonography in trauma (e-FAST)	113	91	22
Lung ultrasound	169	130	39
Abdominal aorta ultrasound	59	54	5
Vascular access – arterial or venous	155	115	40
Fracture identification	13	13	0
Regional anaesthetic delivery	44	42	2
**I find pre-hospital ultrasound clinically?**			
Extremely useful	33	20	13
Very useful	71	43	28
Somewhat useful	104	73	31
Not so useful	18	14	4
Not at all useful	6	6	0
I don't use pre-hospital ultrasound	33	20	13
**In my opinion the benefits of pre-hospital ultrasound include the following?**			
Guides and enhances clinical management	196	123	73

Expediate clinical diagnosis	131	91	40
Helps us to monitor patients	55	32	23
Facilitates interventional procedures	162	110	52
Decreases time to clinical interventions	40	26	14
Enables cognitive offloading	42	30	12
I don't see a benefit of pre-hospital ultrasound	0	0	0
**In my opinion the barriers to pre-hospital ultrasound are as follows?**			
That it does not affect clinical management	69	53	16
Difficult to perform in the clinical environments	97	63	34
The equipment provided is not to sufficient standard	60	37	23
That it delays patient care	92	61	31
That there is a lack of governance associated with ultrasound in a pre-hospital setting	123	76	47
That there is a lack of training standards and accreditation associated with ultrasound in a pre-hospital setting.	28	14	5
I don't feel there are any barriers to pre-hospital ultrasound use	77	62	15

That there is a lack of evidence to show benefit of pre-hospital ultrasound use	100	80	20
